# High-volumetric-capacitance graphene supercapacitors via DEGDE-mediated GO clay process

**DOI:** 10.3389/fchem.2026.1966349

**Published:** 2026-10-12

**Authors:** Jiawen Wu, Dingtao Cheng, Qiuyan Li, Zhihong Wang, Zhou Li, Yuanyuan Li, Yingru Li

**Affiliations:** 1 College of Intelligent Systems Science and Engineering, Hubei Minzu University, Enshi, China; 2 Materials Science and Engineering, Wuhan Institute of Technology, Wuhan, China; 3 School of Chemistry and Environmental Engineering, Hubei Minzu University, Enshi, China; 4 Jianli High School, Jianli, China; 5 Wuhan Wansu Chemical Co., Ltd, Wuhan, China; 6 Key Laboratory of Green Manufacturing of Super-Light Elastomer Materials of State Ethnic Affairs Commission, Hubei Minzu University, Enshi, China

**Keywords:** DEGDE, graphene oxide, reduced graphene oxide, supercapacitor, volumetric capacitance

## Abstract

A refined and facile strategy is reported for preparing high-volumetric-capacitance graphene supercapacitor electrodes via a diethylene glycol dimethyl ether (DEGDE)-mediated graphene oxide (GO) clay process. Building upon the GO “pottery” concept from our previous work, this method addresses the critical limitation of solvent evaporation and loss of workability by employing DEGDE—a high-boiling-point, high-molecular-weight organic solvent—as a processing aid. This modification preserves the plasticity and uniformity of the GO clay during calendering and thermal reduction, enabling the direct production of dense, self-supporting reduced graphene oxide (rGO) electrode sheets without binders, conductive additives, or extensive purification. The resulting rGO electrodes exhibit a well-defined layered graphene architecture with high packing density. In a symmetric two-electrode configuration with 5 M KOH electrolyte, the electrode delivers a gravimetric specific capacitance of 119.4 ± 4.4 F g^‒1^ and a volumetric specific capacitance of 84.0 ± 3.1 F cm^‒3^ at 1 mA cm^‒2^, with stable performance across varying currents and excellent reproducibility. This work provides a practical and cost-effective route for high-density graphene electrodes with competitive volumetric performance for compact energy storage applications.

## 1 Introduction

As global energy demand rises and environmental challenges intensify, developing efficient and eco-friendly energy storage technologies has become a pressing priority (Celadon et al., 2024; Cherp et al., 2011). Among various options, supercapacitors (SCs) have garnered significant attention as a complementary technology to batteries, owing to their exceptional power density, rapid charge/discharge capability, and ultra-long cycle life (Kumar et al., 2022; Li et al., 2019; Oyedotun et al., 2023). Based on their charge storage mechanism, SCs are primarily categorized into electrical double-layer capacitors (EDLCs), which rely on electrostatic adsorption of ions, and pseudocapacitors, which involve fast surface redox reactions (Abdelfatah et al., 2020; Karamanova et al., 2021; Jiang and Liu, 2019; Shah et al., 2023; Vanitha et al., 2026). Pseudocapacitive and battery-type transition-metal oxide/sulfide electrodes, such as Zr-doped NiMnO_3_@NiCoMnS_4_ heterostructures and Ni/Co/Mn multi-metal oxide honeycombs, have achieved high specific capacitances through reversible redox reactions (Mu et al., 2026; Yu et al., 2026). However, carbon-based EDLCs still offer advantages in power density, rate capability, and cycling stability, which motivates the development of graphene-based electrodes. The performance of SCs, especially EDLCs, is fundamentally governed by the physicochemical properties of their electrode materials (Choi et al., 2024; Kadam et al., 2026; Lim et al., 2023). Ideal EDLC electrodes require a high accessible specific surface area for ion adsorption, excellent electrical conductivity for charge transport, a suitable porous structure for electrolyte infiltration, and robust electrochemical stability (Oyedotun et al., 2023; Karamanova et al., 2021; Choi et al., 2024).

Graphene, a two-dimensional material renowned for its exceptional electrical conductivity, exceptionally high theoretical specific surface area, and mechanical robustness, has been widely investigated as a promising electrode candidate for EDLCs (Chang and Hu, 2019; Pathak et al., 2025; Chen et al., 2013; Stoller et al., 2008; Tene et al., 2024). Among graphene-based architectures, three-dimensional graphene aerogels and hydrogels have been extensively explored because their porous networks provide abundant ion-accessible surfaces (Gong et al., 2025). In practice, the focus often centers on its chemically converted derivatives, particularly reduced graphene oxide (rGO), which offer a more feasible route for cost-effective, large-scale production while retaining key properties of pristine graphene (Razaq et al., 2022; Li et al., 2020; Pham et al., 2011). Consequently, various strategies have been developed to architect rGO into functional EDLC electrodes, such as constructing three-dimensional porous hydrogel networks (Sawant et al., 2025; Zhang and Shi, 2011; Ding et al., 2024), employing electrochemical deposition (Sheng et al., 2012; Chang et al., 2012; Li et al., 2013), and utilizing vacuum filtration (Zhang et al., 2012; Yang and Zou, 2014; Wu et al., 2017). While these methods can yield materials with good gravimetric performance, they often involve multi-step processes, require stringent drying conditions, or depend on additional binders and conductive agents. These complexities pose challenges for scalable production and, more critically, often result in electrodes with low packing density, which severely limits their volumetric capacitance—a key metric for practical energy storage devices (Jiang et al., 2015; Elsherif et al., 2022; Yu et al., 2022; Wang et al., 2016). Our previous work pioneered a “graphene oxide mud (GOM)” approach inspired by traditional pottery manufacturing, as GO possesses a lamellar structure with hydrophilic functional groups, resembling clay, and GOM could be readily processed into various shapes (Li et al., 2015). After thermal treatment, the GOM turned into porous graphene monoliths with impressive gravimetric and volumetric capacitances (∼200 F g^‒1^ and ∼200 F cm^‒3^). This approach established the viability of processing GO without extensive purification—a significant step toward simplified and eco-friendly production. However, the reliance on hexane—a low-boiling-point, hydrophobic solvent—led to rapid solvent evaporation and unavoidable water loss during processing. This limitation compromised the workability of the GO clay mud and constrained the precision and scalability of electrode fabrication.

Therefore, building upon the conceptual foundation of GO “pottery” established in our earlier work while addressing its key processing limitation, this study proposes a refined fabrication strategy based on a high-boiling-point-solvent-mediated and moldable “GO clay pellet” process. By employing diethylene glycol dimethyl ether (DEGDE), a high-molecular-weight, high-boiling-point organic solvent, this approach effectively preserves clay plasticity during processing, enabling the direct production of high-density rGO electrodes with enhanced volumetric performance and simplified manufacturability.

## 2 Experimental section

### 2.1 Materials

Graphite powder (325 mesh) was purchased from Qingdao Huatai Lubrication Seal Co., Ltd. Sulfuric acid (H_2_SO_4_), hydrochloric acid (HCl), potassium permanganate (KMnO_4_), hydrogen peroxide (H_2_O_2_, 30%), diethylene glycol dimethyl ether (DEGDE, C_6_H_14_O_3_), and sodium hydroxide (NaOH) were of analytical grade and obtained from Sinopharm Chemical Reagent Co., Ltd. Supercapacitor separators were supplied by Dongguan Hongluo Insulating Material Co., Ltd. All reagents were used as received without further purification.

### 2.2 Synthesis of graphene oxide

Graphene oxide (GO) was synthesized from graphite powder via a modified Hummers’ method (Chen et al., 2015; Zhao et al., 2024). In a typical procedure, 12 g of KMnO_4_ was slowly added to 90 mL of concentrated H_2_SO_4_ in an ice bath under magnetic stirring, maintaining the temperature below 10 °C. After the solution turned dark green, 3 g of graphite powder was introduced, and the mixture was heated to 40 °C with stirring for 1 h. Subsequently, 500 mL of deionized water was rapidly added, followed by dropwise addition of excess H_2_O_2_ until the solution turned bright yellow. The suspension was filtered, and the resulting yellow cake was washed with dilute HCl and deionized water. The crude GO filter cake was freeze-dried and ground into powders, denoted as “GO clay”. Notably, the sonication, centrifugation, and dialysis steps commonly employed in conventional GO purification were omitted, as previously demonstrated to be unnecessary for achieving satisfactory electrode performance (Chen et al., 2015).

### 2.3 Preparation of graphene oxide clay mud and electrodes

The illustration of the fabrication process of graphene electrodes is exhibited in Figure 1a. Typically, the GO clay powders (3 g) was mixed with a solvent system consisting of DEGDE (6 g) and deionized water (1 g) and thoroughly kneaded into a homogeneous clay mud. The resulting GO clay mud was sealed and aged at room temperature for 4 h to ensure uniformity. The mud was then wrapped in aluminum foil and rolled into sheet using a manual roller press. Circular electrode discs (1 cm in diameter) were punched from the rolled sheets and thermally reduced at 180 °C for 6 h in a drying oven. After cooling to room temperature, the aluminum foil was removed, and the resulting reduced graphene oxide (rGO) electrode discs were washed three times with hot deionized water (80 °C) to remove residual impurities and DEGDE, yielding the final graphene electrodes (denoted as GE).

**FIGURE 1:**
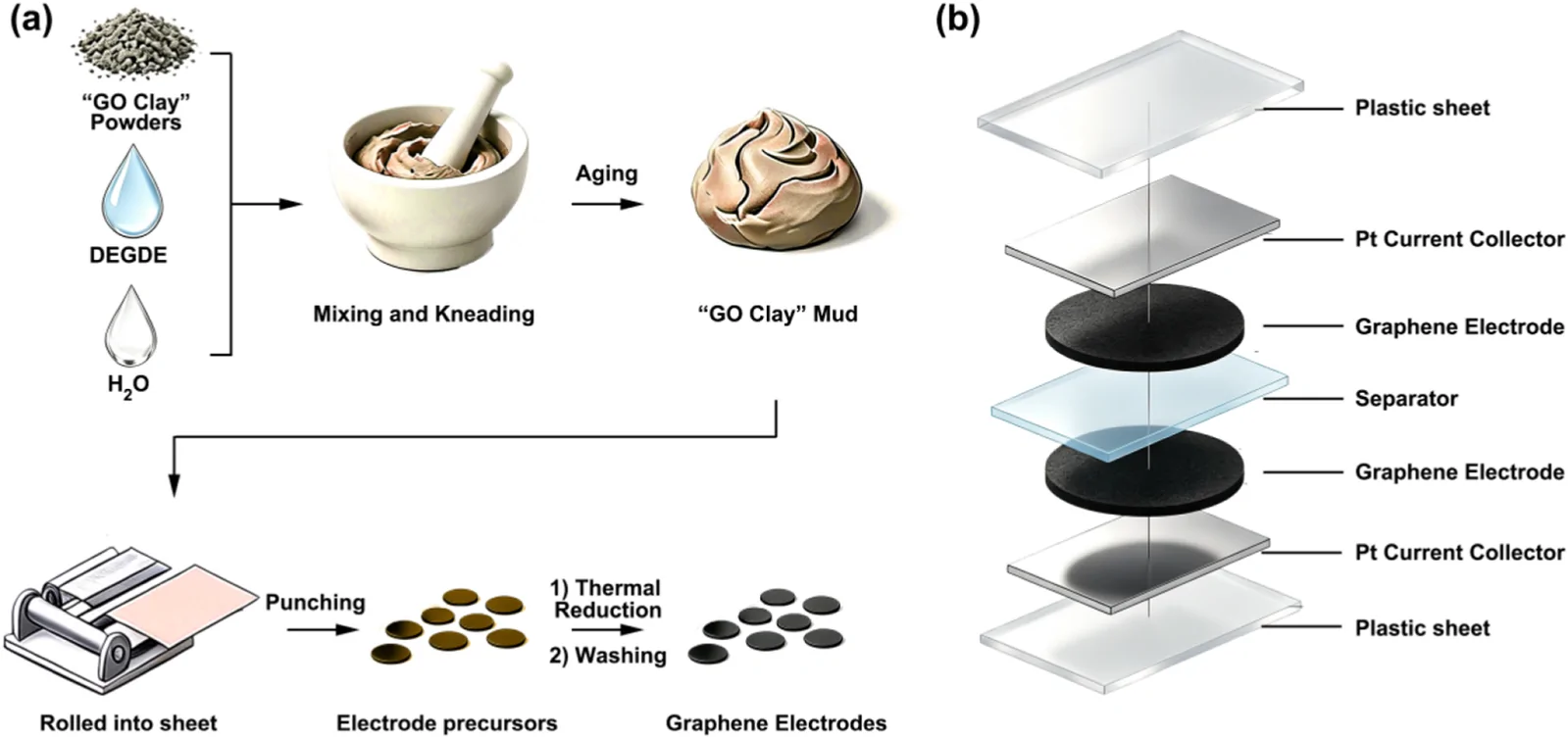
**(a)** Schematic illustration of the fabrication process of graphene electrodes (GE) via the DEGDE-mediated GO clay mud route. **(b)** Schematic diagram of the assembled symmetric two-electrode supercapacitor configuration.

### 2.4 Assembly of two-electrode supercapacitors

The GE electrode discs and separator membranes were immersed in 5 M KOH aqueous solution for 24 h prior to assembly. Symmetric two-electrode supercapacitors were assembled in a bottom-up sequence: a plastic sheet, a platinum plate, an electrode, a separator, another electrode, another platinum plate, another plastic sheet (Figure 1b), with careful alignment of the two electrodes.

### 2.5 Material characterization

Morphologies of the GE samples were examined using scanning electron microscopy (SEM, ZEISS GeminiSEM 300, Germany) (Tene et al., 2024). Raman spectra were recorded on a Horiba LabRAM HR Evolution system (Japan) with a 532 nm laser at 4.7 mW (Tene et al., 2024). X-ray photoelectron spectroscopy (XPS) was performed on a Thermo Scientific ESCALAB Xi instrument with monochromatic Al Kα radiation (Li et al., 2015).

### 2.6 Electrochemical measurements

Electrochemical tests were conducted on a CHI 760E electrochemical workstation (Shanghai Chenhua, China) using a two-electrode system. Cyclic voltammetry (CV) was performed at scan rates of 1, 2, 5, and 10 mV s^‒1^ in the voltage range of 0–1 V. Galvanostatic charge-discharge (GCD) measurements were conducted at currents of 1, 2, 5, and 10 mA cm^‒2^ within the same voltage window. Electrochemical impedance spectroscopy (EIS) was carried out at open-circuit potential with a frequency range of 100 kHz to 0.01 Hz and an AC amplitude of 5 mV. All tests were performed at room temperature using 5 M KOH as the electrolyte.

### 2.7 Calculation of specific capacitance

The specific capacitance of the assembled supercapacitor was calculated from the GCD curves. The cell capacitance was determined according to:

$$C_{cell}=\frac{I\Delta t}{\Delta U}$$ (1)

where *I* is the discharge current (A), Δt is the discharge time (s), ΔU is the voltage change during discharge excluding *iR* drop (V) and *C*_*cell*_ is the cell capacitance (F). For the symmetric two-electrode configuration, the capacitance of a single electrode was calculated as:

$$C=2C_{cell}$$ (2)

where *C* is the capacitance of a single electrode (F). The gravimetric specific capacitance and volumetric specific capacitance of the single electrode were then calculated as:

$$C_{m}=\frac{C}{m},C_{V}=\frac{C}{V}$$ (3)

where *C*_*m*_ is the gravimetric specific capacitance (F g^−1^), *C*_*v*_ is the volumetric specific capacitance (F cm^−3^), m is the mass of a single electrode disc (g), and *V* is the volume of a single electrode disc (cm^3^). The electrode area was 0.785 cm^2^ (1 cm in diameter). The current density (mA cm^−2^) was converted to the discharge current *I* (A) by multiplying by the electrode area. The electrode volume was determined from the disc diameter (1 cm) and thickness measured by SEM cross-sectional imaging.

## 3 Results and discussion

### 3.1 Morphological characterization

The digital photographs of the as-prepared GO clay mud and the final GE are shown in Figure 2. The GO clay mud, obtained by kneading GO clay powder with DEGDE and deionized water, exhibits a brownish, lumpy paste with a smooth and cohesive texture, indicating good plasticity and processability (Figure 2a). After rolling, punching, and thermal reduction at 180 °C for 6 h, the resulting GE disc presents a shiny black, circular sheet with a diameter of 1 cm and a well-defined, crack-free surface, suggesting successful compaction and reduction without structural disintegration (Figure 2b).

**FIGURE 2:**
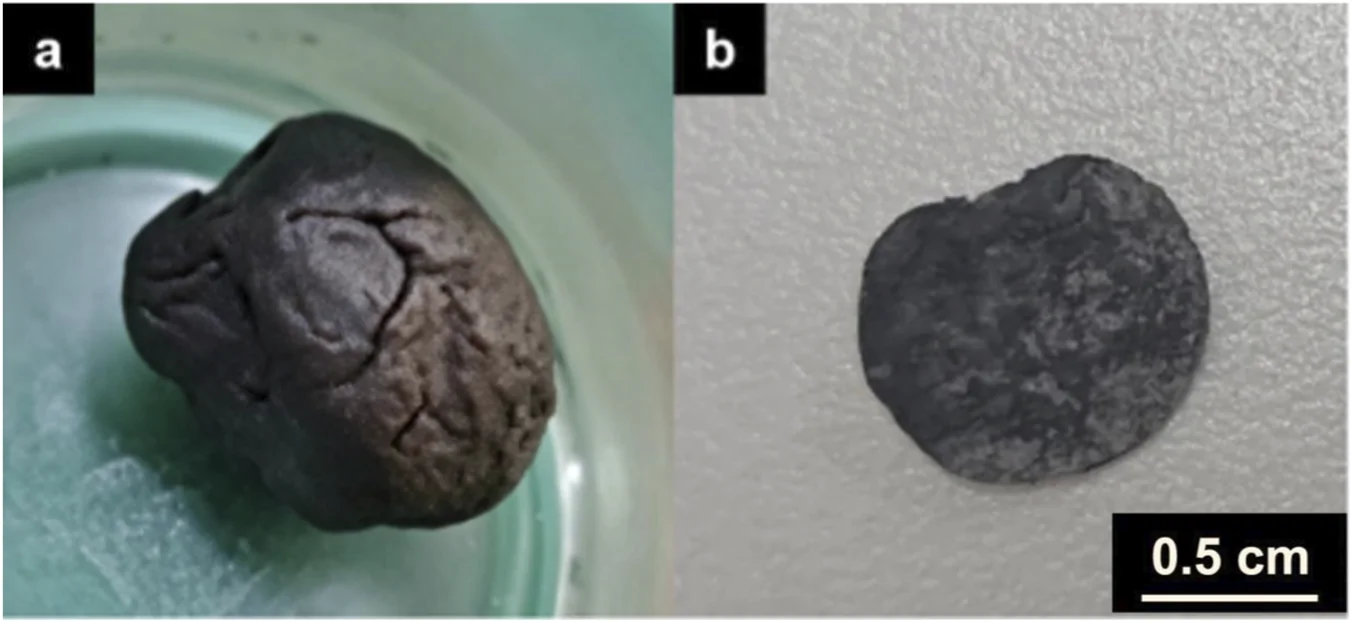
Digital photographs of the as-prepared GO clay mud **(a)** and the final GE **(b)**.

The internal microstructure of the GE was further characterized by cross-sectional SEM at various magnifications (Figure 3). The low-magnification images (Figures 3a,b) reveal a compact and uniform internal structure throughout the electrode thickness, with no significant voids, cracks, or delamination observed. The electrode thickness is measured from Figures 3b to be approximately 120 μm, enabling the subsequent calculation of volumetric capacitance. At higher magnifications (Figures 3c,d), the lamellar architecture characteristic of reduced graphene oxide sheets is clearly resolved. The cross-section exhibits densely packed rGO layers with fine interlayer spaces and occasional wrinkled features, indicating that the calendering process effectively aligned the graphene sheets while preserving sufficient porosity for electrolyte infiltration.

**FIGURE 3:**
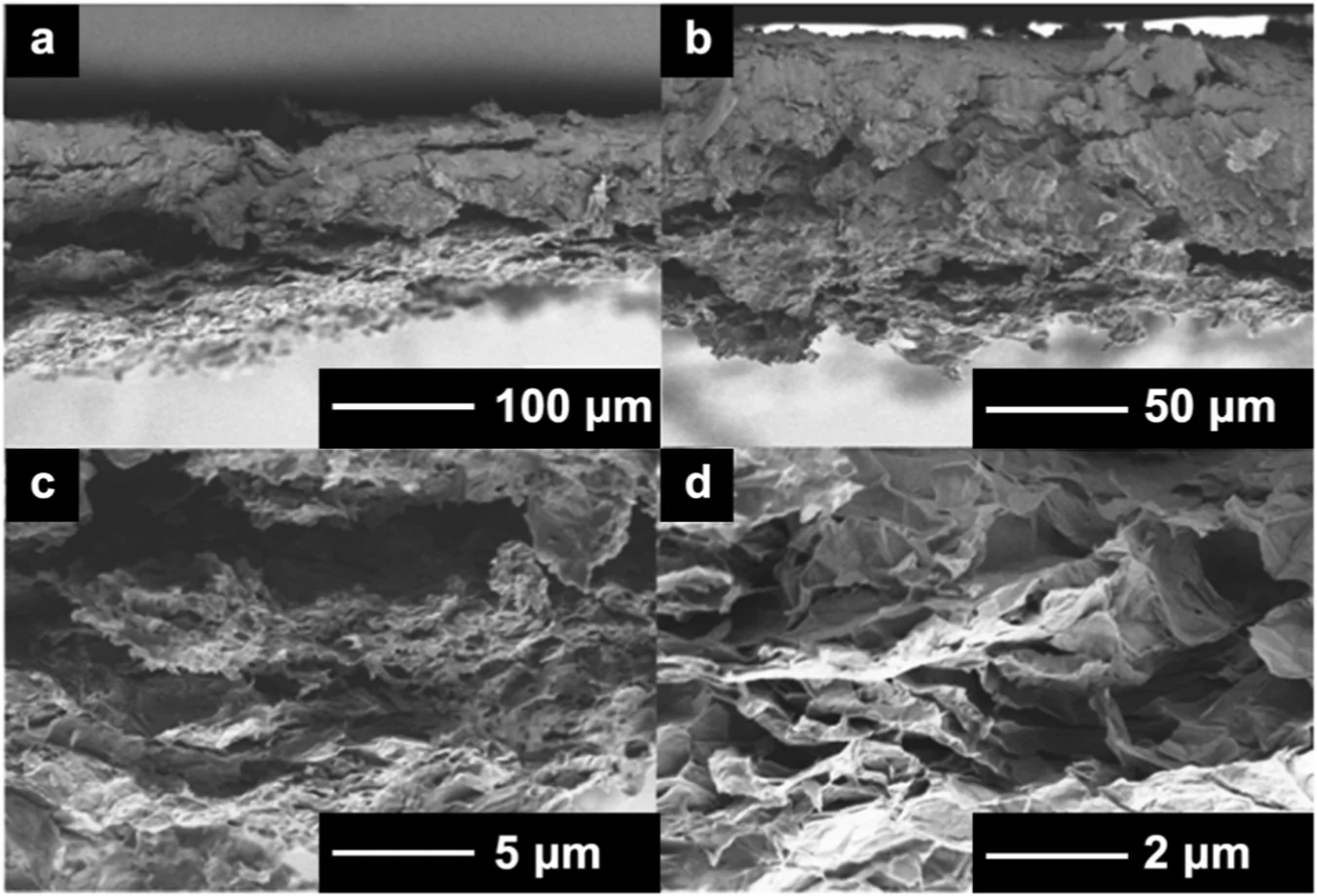
Cross-sectional SEM images of GE at different magnifications. Scale bars: **(a)** 100 μm, **(b)** 50 μm, **(c)** 5 μm, **(d)** 2 μm.

The compact yet porous architecture observed in SEM cross-sections is attributed to the dual role of DEGDE: its high boiling point (162 °C) preserves the plasticity of the GO clay mud during calendering, while its gradual evaporation during thermal reduction creates interconnected pores within the rGO matrix. This balanced microstructure is dense enough for high volumetric packing, yet porous enough for electrolyte infiltration. It is central to the electrochemical performance discussed below.

### 3.2 Chemical structural analysis

The chemical structures of GO and GE were investigated by XPS and Raman spectroscopy (Chen et al., 2015; Zhao et al., 2024). Quantitative analysis based on the XPS survey spectra (data summarized in Table 1) reveals that the apparent oxygen content of the as-prepared GO is as high as 45.51 at% (with carbon at 46.78 at%), giving an apparent C/O ratio of approximately 1:1. This value is anomalously low compared to typical GO, and is primarily attributed to the substantial residual sulfate and sulfate ester species (7.63 at% S) introduced during the Hummers’ oxidation, where each sulfur atom coordinates with four oxygen atoms. After thermal reduction and washing, the sulfur content is significantly reduced to 0.70 at%, and the oxygen content decreases to 12.11 at%, while carbon increases to 87.07 at%.

**TABLE 1:** Elemental composition of GO and GE determined by XPS survey spectra.

Element	C (at%)	O (at%)	S (at%)	Mn (at%)	C/O ratio (apparent)	C/O ratio (corrected)*
GO	46.78	45.51	7.63	0.08	∼1.0:1	∼3.1:1
GE	87.07	12.11	0.70	0.12	∼7.2:1	∼9.4:1

* Corrected by excluding oxygen atoms associated with sulfate/sulfate ester species (based on the stoichiometry of –OSO_3_H, i.e., four O atoms per S atom).

After excluding the oxygen atoms associated with sulfur (based on the stoichiometry of –OSO_3_H, i.e., four O atoms per S atom) (Li et al., 2015), the corrected C/O ratio of the GO backbone is calculated to be approximately 3.1:1, which falls within the typical range reported for graphene oxide prepared by the Hummers’ method. For GE, the corrected C/O ratio increases to approximately 9.4:1, confirming the effective removal of both oxygen-containing functional groups and sulfur-containing impurities. Manganese signals in both samples are barely distinguishable from the background noise (Table 1, Mn: 0.08 at% for GO and 0.12 at% for GE), indicating that residual KMnO_4_ and its reduction byproducts were effectively removed during the washing process of the crude GO filter cake with dilute HCl and deionized water.

The high-resolution C1s spectra provide further insight into the chemical transformations. The C1s spectrum of GO (Figure 4a) can be deconvoluted into three peaks corresponding to C–C/C=C (284.6 eV), C–O (286.6 eV), and C=O (287.8 eV), indicative of the extensive oxidation of graphite. After thermal reduction, the GE C1s spectrum (Figure 4b) exhibits peaks at C–C/C=C (284.6 eV), C–O (286.6 eV), and O–C=O (289.0 eV) (Tene et al., 2024; Li et al., 2015). The disappearance of the C=O signal and the emergence of O–C=O suggest that carbonyl groups were partially transformed into carboxylate configurations during thermal treatment, while the overall intensity of oxygen-related peaks diminished substantially.

**FIGURE 4:**
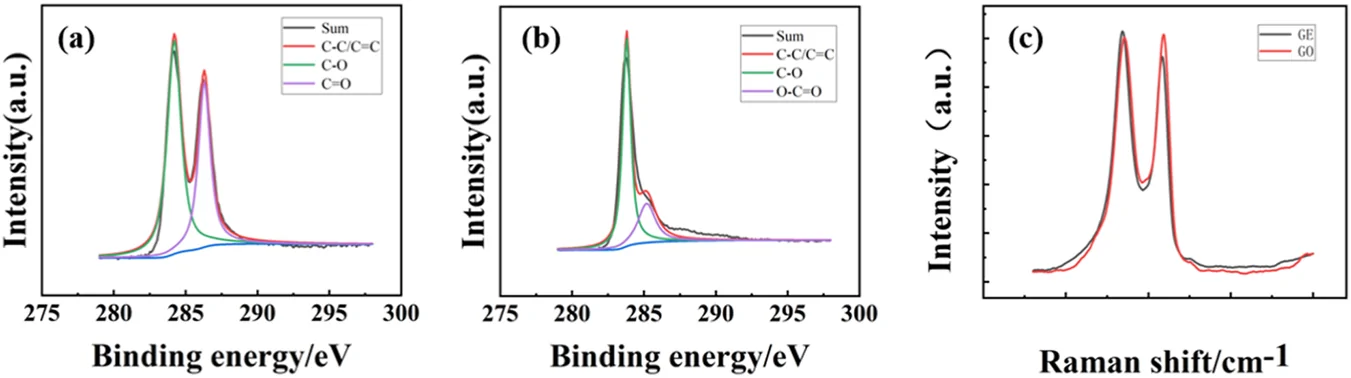
**(a)** High-resolution XPS C1s spectrum of GO; **(b)** high-resolution XPS C1s spectrum of GE; **(c)** Raman spectra of GO and GE (excitation wavelength: 532 nm).

Raman spectroscopy was employed to further evaluate the structural features (Figure 4c). Both GO and GE exhibit two characteristic bands: the D band at ∼1,350 cm^‒1^, originating from structural defects and disordered carbon domains, and the G band at ∼1,590 cm^‒1^, corresponding to the in-plane vibration of sp^2^ carbon atoms (Li et al., 2013; Zhang et al., 2012). The intensity ratio I_D_/I_G_ increased from 0.93 for GO to 1.08 for GE, indicating the creation of more defects or smaller graphitic domains upon reduction—a phenomenon commonly observed in thermally reduced graphene oxides, attributable to the removal of oxygen functional groups and the accompanying structural rearrangement.

In summary, the morphological and structural analyses above establish two key features of the GE material. First, the compact, lamellar microstructure with well-aligned rGO sheets and a thickness of ∼120 μm provides a dense yet accessible electrode architecture, which is essential for achieving high volumetric capacitance. Second, the effective thermal reduction (C/O ratio increased from 3.1:1 to 9.4:1) and successful removal of sulfur and manganese impurities ensure a clean carbon framework with restored sp^2^ domains and moderate defects, favoring both electrical conductivity and ion adsorption. These characteristics, combined with the simplified processing enabled by the DEGDE-mediated clay mud approach, position GE as a promising candidate for high-performance, practically feasible supercapacitor electrodes.

### 3.3 Electrochemical performance

#### 3.3.1 Cyclic voltammetry

CV curves of the GE electrode were recorded at scan rates ranging from 1 to 10 mV s^‒1^ within a potential window of 0–1 V (Figure 5). All CV curves exhibit a quasi-rectangular shape, characteristic of ideal EDLC behavior, with no distinct redox peaks, indicating that the charge storage is dominated by electrostatic double-layer capacitance (Zhang et al., 2012). As the scan rate increases from 1 to 10 mV s^‒1^, the CV curves retain their rectangular profile with minor distortion, suggesting good rate capability and rapid ion transport within the electrode structure. The slight deviation from perfect rectangularity at higher scan rates can be attributed to the internal resistance of the electrode and the diffusion limitations of electrolyte ions within the compact layered structure.

**FIGURE 5:**
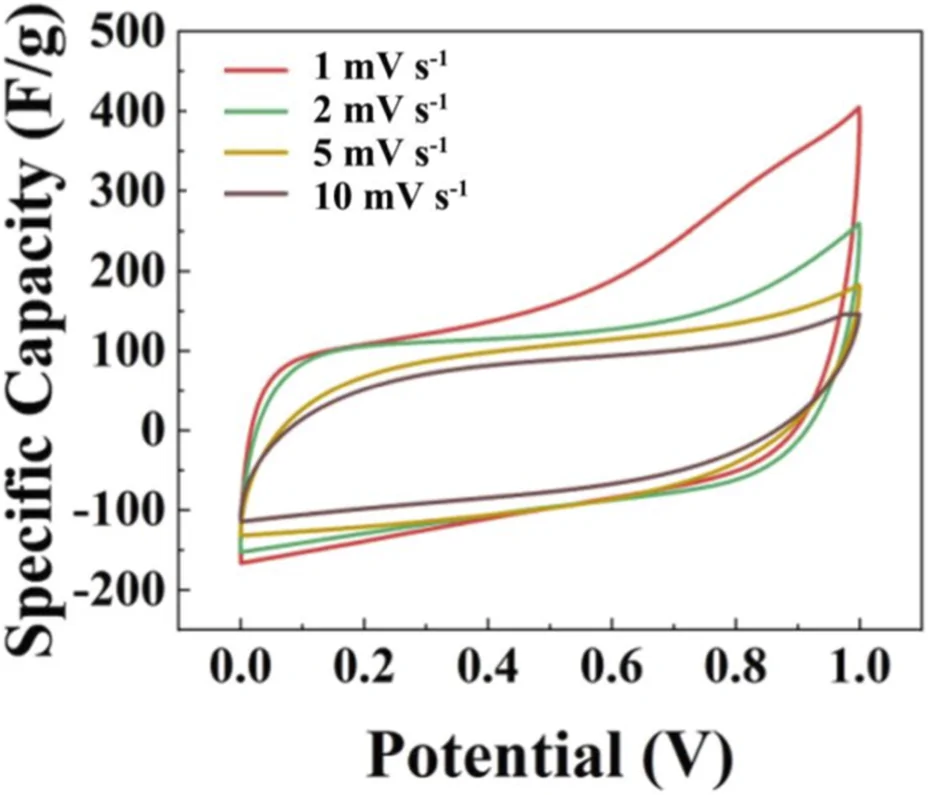
Cyclic voltammetry curves of GE at different scan rates (1, 2, 5, and 10 mV s^‒1^) in the potential range of 0–1 V.

#### 3.3.2 Galvanostatic charge-discharge

GCD measurements were performed at currents of 1, 2, 5, and 10 mA cm^‒2^ within the same potential window (Figure 6). All GCD curves display nearly symmetric triangular profiles with linear charge and discharge segments, confirming the reversible and capacitive nature of the charge storage process. The slight curvature observed at the beginning of the discharge curves is attributed to the internal resistance (*iR* drop) of the assembled cell. Notably, the triangular shapes are well maintained even at the highest current (10 mA cm^‒2^), demonstrating good rate performance.

**FIGURE 6:**
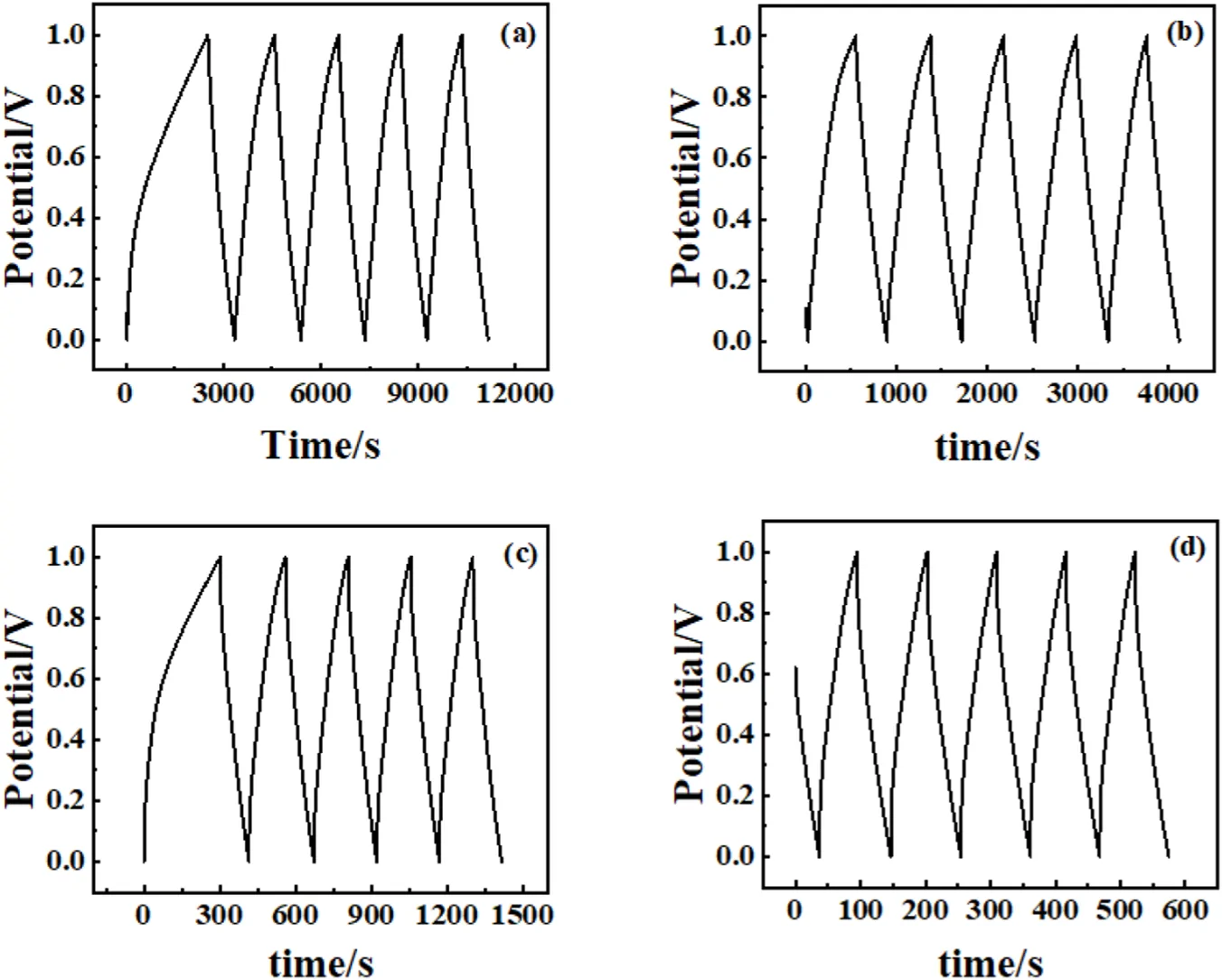
Galvanostatic charge-discharge curves of GE at different current densities in the potential range of 0–1 V: **(a)** 1 mA cm^‒2^, **(b)** 2 mA cm^‒2^, **(c)** 5 mA cm^‒2^, **(d)** 10 mA cm^‒2^.

The specific capacitances calculated based on Equations 1–3 from the GCD curves are summarized in Table 2. At 1 mA cm^‒2^, the GE electrode achieves a gravimetric specific capacitance of 119.4 ± 4.4 F g^‒1^ and a volumetric specific capacitance of 84.0 ± 3.1 F cm^‒3^. As the discharge current increases to 10 mA cm^‒2^, the specific capacitance decreases to 74.7 ± 10.8 F g^‒1^ and 52.5 ± 7.6 F cm^‒3^, respectively. This decay is anticipated for carbon-based EDLC electrodes, as higher currents limit the time available for ion diffusion into the electrode interior, resulting in reduced utilization of the accessible surface area. The rate capability was evaluated based on the specific capacitances at different current densities. The capacitance retention from 1 to 10 mA cm^‒2^ is approximately 63%, indicating good rate performance. The relatively small standard deviations across parallel measurements confirm the good reproducibility of the fabrication protocol.

**TABLE 2:** Gravimetric and volumetric specific capacitances of GE at different current densities.

Current density	10 mA cm^‒2^	5 mA cm^‒2^	2 mA cm^‒2^	1 mA cm^‒2^
Cm (F g^‒1^)	74.7 ± 10.8	90.1 ± 2.3	106.5 ± 0.9	119.4 ± 4.4
C_V_ (F cm^‒3^)	52.5 ± 7.6	63.3 ± 1.6	74.8 ± 0.5	84.0 ± 3.1

It is worth noting that the gravimetric capacitance of GE (∼119.4 F g^‒1^) is lower than that of our previously reported GOM-derived porous graphene monoliths (∼200 F g^‒1^) (Li et al., 2015), as well as some low-density graphene aerogels or hydrogels reported in the literature. This reduction is a direct consequence of the deliberate processing choices made in the present study: the calendering step, while essential for achieving high packing density and mechanical integrity, inevitably reduces the specific surface area available for ion adsorption by compacting the rGO sheets. In addition, dialysis, centrifugation, and sonication steps were eliminated. This simplification reduces production costs but may leave a fraction of residual impurities and some sheet aggregation, both of which can moderately suppress the gravimetric performance.

However, this trade-off is justified when considering the metrics that truly matter for practical applications. The volumetric capacitance of 84.0 ± 3.1 F cm^‒3^ achieved here compares favorably with many rGO-based electrodes prepared by more complex methods, and is realized through a process that requires only simple kneading, rolling, and thermal treatment, without binders, conductive additives, or extensive purification. For compact energy storage devices where available volume is limited, this combination of competitive volumetric performance and processing simplicity represents a meaningful advance toward practical feasibility.

#### 3.3.3 Electrochemical impedance spectroscopy

EIS measurements were conducted to investigate the ion transport behavior and internal resistance of the GE electrode. The Nyquist plot (Figure 7) exhibits a semicircle in the high-frequency region followed by a straight line in the low-frequency region, agreeing with the performance of porous electrode (de Levie, 1963). The high-frequency intercept with the real axis corresponds to the equivalent series resistance (ESR), determined to be approximately 3.5 Ω, primarily arising from the electrolyte resistance and contact resistance at the electrode–current collector interface. The small semicircle diameter suggests a relatively low charge-transfer resistance, indicating good electrical conductivity of the GE electrode.

**FIGURE 7:**
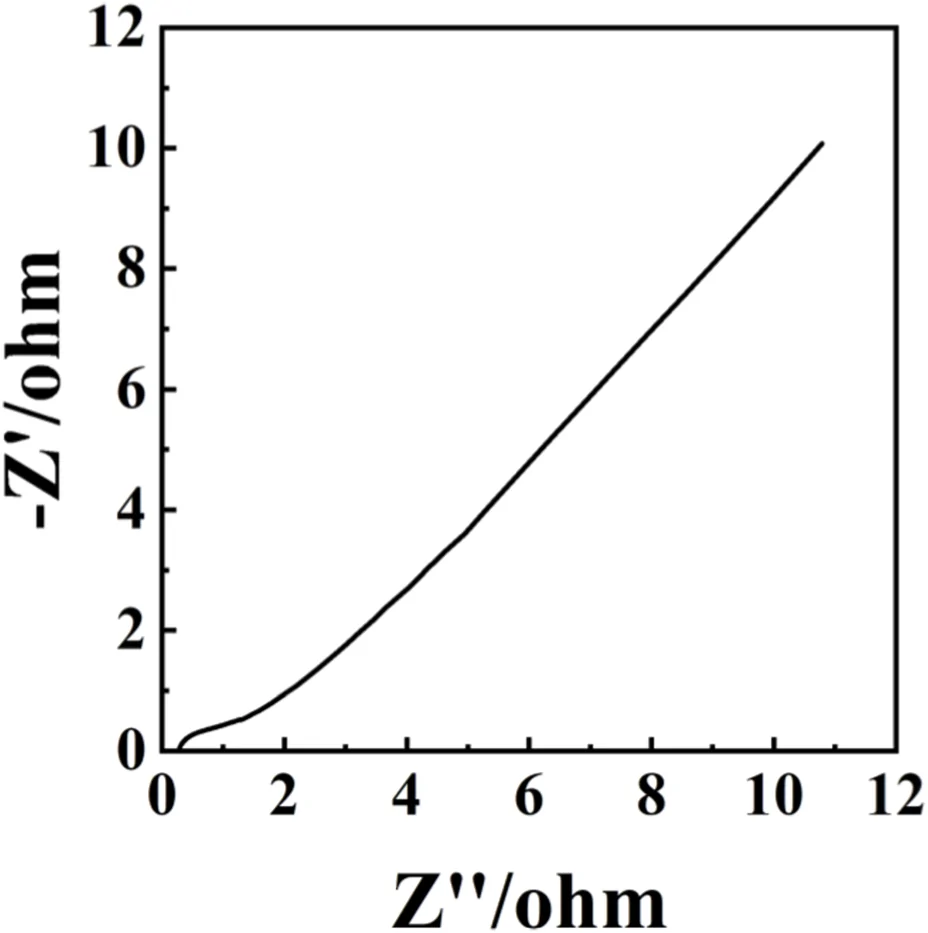
Nyquist plot of the GE-based supercapacitor in the frequency range of 100 kHz to 0.01 Hz with an AC amplitude of 5 mV.

In the low-frequency region, the nearly vertical straight line oriented at an angle of approximately 45° to the real axis is characteristic of porous electrodes, as described by the de Levie model (de Levie, 1963), where ion diffusion within the porous structure contributes to the impedance. The deviation from an ideal vertical line (which would be expected for a purely capacitive electrode) suggests the presence of distributed pore sizes and some degree of diffusion limitation within the compact electrode architecture. Nonetheless, the overall EIS response indicates good capacitive behavior with favorable ion accessibility.

### 3.4 Discussion

The electrochemical performance of GE reflects a deliberate trade-off between gravimetric and volumetric metrics. The gravimetric capacitance (∼119 F g^‒1^) is lower than that of our previously reported porous graphene monoliths (∼200 F g^‒1^), as well as some low-density graphene aerogels or hydrogels in the literature. This reduction arises from the calendering step, which compacts rGO sheets into a dense architecture, and the omission of dialysis, centrifugation, and sonication—both choices that prioritize process simplicity and volumetric efficiency over maximizing specific surface area.

Nevertheless, the volumetric capacitance of 84.0 F cm^‒3^ achieved here is competitive with many rGO-based electrodes prepared by more complex methods. More importantly, this performance is realized through a process that requires only kneading, rolling, and thermal treatment—without binders, conductive additives, or extensive purification. For compact energy storage devices where available volume is limited and manufacturing cost matters, this balance between performance and practical feasibility represents a meaningful advance.

## 4 Conclusion

In summary, this study demonstrates a refined and simplified strategy for the fabrication of high-volumetric-capacitance graphene supercapacitor electrodes through a DEGDE-mediated GO clay mud process. By substituting the volatile hexane used in our previous work with a high-boiling-point solvent (DEGDE), the method effectively preserves the plasticity and workability of the GO clay during calendering and thermal reduction, enabling the production of dense, crack-free, and self-supporting rGO electrode sheets without binders, conductive additives, or extensive purification. The resulting GE electrodes exhibit well-defined layered architectures with high packing density, achieving a gravimetric specific capacitance of 119.4 ± 4.4 F g^‒1^ and a competitive volumetric capacitance of 84.0 ± 3.1 F cm^‒3^ at 1 mA cm^‒2^ with good rate capability and reproducibility. The gravimetric performance is moderate compared to low-density graphene aerogels. However, this trade-off is justified by the superior volumetric performance and by the exceptional process simplicity. The method eliminates dialysis, centrifugation, and sonication steps and uses only commercially available reagents and standard equipment. This balance between performance and practical feasibility positions the DEGDE-mediated clay mud strategy as a promising route toward scalable, cost-effective production of high-density graphene electrodes for compact energy storage applications.

## Data Availability

The raw data supporting the conclusions of this article will be made available by the authors, without undue reservation.
